# Protection of patients against violence in medical institutions and the need for general safeguarding measures

**DOI:** 10.1007/s00787-022-02007-5

**Published:** 2022-06-20

**Authors:** Ulrike Hoffmann, C. G. Svedin, D. Anagnostopoulus, J. P. Raynaud, A. M. Räberg Christensen, Jörg M. Fegert

**Affiliations:** 1 University Hospital, Ulm, Germany; 2 Marie Cederschiöld University, Stockholm, Sweden; 3 National and Kapodistrian University of Athens, Athen, Greece; 4 Toulouse University Hospital, Toulouse, France; 5 Child and Adolescent Psychiatric Center, Glostrup, Denmark

In the last decades, sexual abuse and violence in institutions against minors has been a repeated topic of discussion in politics and among experts in the field in Germany. The alleged abuse scandal in 2010 marks a central event. At that time, sexual abuse in institutions became public again starting in January 2010. This was mainly due to the confession made by the Canisius College in Berlin admitting that cases of sexual abuse had been covered up in the institution for years. Subsequently, cases also became known from other Catholic institutions. At the end of February 2010, it was reveiled that the former director had sexually abused pupils during his years of service at the Odenwaldschule Oberhambach, which was one of the flagship projects of reform pedagogy. In the course of 2010, other teachers of this school and other institutions of reform pedagogy were accused of having committed sexual assaults. These revelations about the scope and severity of past cases of child sexual abuse in German institutions set off a broad public debate. The debate led to the establishment of a politically appointed Round Table committee and an Independent Commissioner whose mandates were to reappraise the issue and develop recommendations for future policies. The Round Table Committee demanded that structural measures be implemented in institutions to protect children and adolescents from (sexual) abuse. In Germany, these measures are summarized as “Schutzkonzepte” (Safeguarding measures). The goal of these measures is to better ensure the protection of children and adolescents from sexual abuse and violence in an institution. In its final report, the Round Table Committee defined components for the safeguarding measures (see Table 1) and recommended the implementation of these measures to all institutions.

**Table 1:** Safeguarding measures against sexual abuse in institutions (UBSKM 2011, adapted version according to Hoffmann et al. 2021)

Subsections	Elements of safeguarding measures
Analysis	Risk analysis Analysis of already existing measures and potential of the institution with regard to child protection/protection against violence
Prevention	Preventive measures for children and adolescents Mission statement Code of conduct/behavioural guidelines Forms of participation for children/adolescents, parents and employees Concept for dealing with complaints and feedback Pedagogical concept, concept for sexual education and media education Child protection-sensitive staff recruitment and development Employment contract regulations, e.g. self-commitment declaration, specific legal regulation (e.g. in Germany extended police clearance certificate) Considering child protection criteria in personnel selection Regular training of employees
Intervention	Concept for dealing with employee misconduct Guidelines for the procedure with (suspected) cases of (sexual) abuse
Dealing with past cases	Recommendations for dealing with past cases (compensation for victims, “learning from mistakes”) Concept for rehabilitation after false accusation

Due to the fact that most cases in 2010 had taken place in the Roman Catholic Church and in the educational field, the medical field is receiving very little focus. In contrast to the ongoing debates about dealing with cases, for instance, in institutions of the Roman Catholic Church or in sports there have been no systematic efforts in learning from past mistakes and from victims of abuse and violence in the health sector. Therefore, for both the public and among professionals working in the medical field, hospitals and medical facilities are seen as places offering protection, help and therapy to vulnerable patients. Unfortunately, the idea that the dependency and the vulnerability of patients could be used by perpetrators for assaults is often ignored. It is not sufficiently recognized that abuse not only happens frequently but also systematically, as well as in other institutional areas, not least because of the lack of reappraisal.

In Germany, the alleged abuse scandal in 2010 helped to raise awareness of the issue of sexual assault in institutions as a whole. It also inspired research surrounding the prevalence of such incidents, including projects exploring how to deal with assaults in institutions and initiatives for the broad implementation of safeguarding measures in institutions. The implementation of political structures such as the Independent Commissioner on Child Sexual Abuse and the Independent Inquiry into Child Sexual Abuse also plays an important role for dealing with the issue on an ongoing basis.

In recent years, research activities have shown the dimension of sexual assault in various types of institutions and have made institutional risk factors and structures promoting abuse visible. However, deficits continue to exist in certain areas, e.g., there are hardly any studies on prevalence of abuse in the medical sector. The few existing studies indicate a high prevalence [2–4]. The prevalence rates in medical institutions are also to be judged differently since in contrast to school, for example, children and adolescents in hospitals only have short durations of stay.

The issue of sexual assault in institutions has also been discussed by professionals in other European countries. In the following, such national debates are exemplarily presented.

In **Sweden**, the findings in a recent review of coercive measures within the social institution area [1] have led to an intense debate about violence and abuse within these institutions. The placed children often have a complex mix of delinquent behavior, substance abuse, mental illness, school and family problems and are to a large extent also similar to the children found in child and adolescent psychiatric round-the-clock care. What is even more concerning is that those worst affected were younger children, girls and children with disabilities. This alarming report has influenced the issue of violence and abuse of children in institutional care to be incorporated in the ongoing Government inquiry “A childhood free from violence”.

In **Denmark**, the social authorities control the social institutions. Additionally, they have recently been involved in a case of severe verbal obscene abuse in one of the institutions. In the mental hospitals, there is a specific ‘psychiatric complaint board’ that takes care of patient’s rights. There is a lot of focus on preventing coercion and—some good initiatives, e.g. Safeward and the ‘Six Core Strategies to prevention’—and some negative consequences in the form of government partnerships demanding to reduce the level of coercion, but not providing the means to do so.

In **Greece**, safeguarding measures and procedures are not yet commonly practiced and implemented. Due to a psychiatric reform, large psychiatric hospitals were closed. The hospitalization of children and adolescents occurs in small units within general paediatric hospitals for a short period of time. As a consequence of this change, the reported cases of maltreatment decreased dramatically. However, this empirical conclusion has not yet been confirmed by research.

Generally, all institutions have risk factors for sexual abuse and other forms of violence. For the medical sector, it must be considered that all patients in medical treatment and psychotherapy have to accept a relationship of dependency, which leaves them relatively vulnerable. Other risk factors specific to the medical field include the patients’ lack of knowledge regarding the necessity and adequacy of medical measures, the lack of knowledge about regular procedures and responsibilities in clinics, and the hierarchies in hospitals.

Certain interventions such as the coercive measures in psychiatry have a particularly high risk of entailing an abuse of power. The field of child and adolescent psychiatry bears some specific risk factors, which includes the emotional and close relationships between professionals and mentally ill children and adolescents, as well as the high proportion of children and adolescents with a history of adverse childhood experiences resulting in an increased risk of being affected again by assaults in institutions.

Due to the different risk factors of the different types of institutions, it is necessary to adapt the already mentioned elements of safeguarding measures such as a mission statement, a complaint system and an intervention plan to the specific institution.

Other aspects must be considered when discussing the responsibility of each institution in preventing the abuse of children and adolescents, which involves the high prevalence of sexual abuse and other forms of violence in the family context. Many children and adolescents affected by someone in their family come into contact with institutions such as schools and the medical sector. Therefore, it is important for institutions to be places where appropriate help and support are provided. In Germany, many large hospitals have already incorporated specific interdisciplinary child protection groups for this purpose. Since 2019, a child protection guideline has been implemented.

In 2015–2018, an evaluation of the implementation of safeguarding measures in various fields of institutions including the medical sector was conducted in Germany on behalf of the Independent Commissioner on Child Sexual Abuse. The results have shown that only a small proportion of medical institutions had already become active in implementing safeguarding measures. The reason for this could have been due to the lack of a binding commitment to implement safeguarding measures. It was not until 2020 that the Federal Joint Committee (G-BA) which is the authority for defining quality management measures in medical institutions in Germany made the implementation of safeguarding measures mandatory and defined minimum obligatory measures. These measures are based on the specifications made by the Round Table on Child Sexual Abuse.

The mandatory nature of these requirements is very much welcomed because developments in Germany have shown two things: first, it became clear that institutions often only address the issue of sexual abuse and violence under the pressure of concrete cases in the institution. Second, in many places, the first step in responding to the problem consisted of presenting the issue to a single specialist, who was then supposed to work through the cases and implement measures in the institution. When measures are developed and implemented in this manner, there is a risk that they will not be practiced in the institution. An important part of developing safeguarding measures is developing an attitude that does not tolerate sexual assault and violence. This type of mindset and culture can only be created and maintained if safeguarding measures are jointly developed in an ongoing process.

The implementation of safeguarding measures requires resources and a long-term commitment from the institutions. Nonetheless, the implementation of these measures is beneficial because discussions regarding the protection of minors against violence in the institution can contribute to a better working environment for every individual. Additionally, it also provides safety for the professionals since these discussions help to clarify how to proceed in specific cases concerning abuse. Furthermore, based on the experience in Germany, we would like to encourage a Europe-wide debate on such safeguarding measures because we consider the creation of a Europe-wide recommendation and its endorsement to be very useful. We would be more than happy to discuss this with colleagues from all European countries and to set up a working group to draw up the recommendations.
